# Shear-Induced Crystallization of Star and Linear Poly(L-lactide)s

**DOI:** 10.3390/molecules26216601

**Published:** 2021-10-31

**Authors:** Joanna Bojda, Ewa Piorkowska, Grzegorz Lapienis, Adam Michalski

**Affiliations:** Centre of Molecular and Macromolecular Studies, Polish Academy of Sciences, Sienkiewicza 112, 90-363 Lodz, Poland; epiorkow@cbmm.lodz.pl (E.P.); lapienis@cbmm.lodz.pl (G.L.); michadam@cbmm.lodz.pl (A.M.)

**Keywords:** poly(L-lactide), star poly(L-lactide), shear-induced crystallization, crystallinity, fibrillar nuclei

## Abstract

The influence of macromolecular architecture on shear-induced crystallization of poly(L-lactide) (PLLA) was studied. To this aim, three star PLLAs, 6-arm with M_w_ of 120 and 245 kg/mol, 4-arm with M_w_ of 123 kg/mol, and three linear PLLAs with M_w_ of 121, 240 and 339 kg/mol, were synthesized and examined. The PLLAs were sheared at 170 and 150 °C, at 5/s, 10/s and 20/s for 20 s, 10 s and 5 s, respectively, and then cooled at 10 or 30 °C/min. Shear-induced crystallization during cooling was followed by a light depolarization method, whereas the crystallized specimens were examined by DSC, 2D-WAXS, 2D-SAXS and SEM. The effect of shear depended on the shearing conditions, cooling rate and polymer molar mass but it was also affected by the macromolecular architecture. The shear-induced crystallization of linear PLLA with M_w_ of 240 kg/mol was more intense than that of the 6-arm polymer with similar M_w_, most possibly due to its higher M_z_. However, the influence of shear on the crystallization of the star polymers with M_w_ close to 120 kg/mol was stronger than on that of their linear analog. This was reflected in higher crystallization temperature, as well as crystallinity achieved during cooling.

## 1. Introduction

In recent decades, bio-based polymers derived from annually renewable resources have drawn increasing attention [1], as biomass is the only source of available renewable carbon. Among them, polylactide (PLA) is the most promising polymer for the replacement of conventional thermoplastics, especially because it is also biodegradable (compostable) and can be used for a wide range of applications, including biomedical products, textiles, daily appliances, packaging, items used in agriculture and engineering [1,2,3,4,5].

Amorphous PLA with the glass transition temperature, T_g_, in the range of 55–60 °C, is stiff and brittle at room temperature. The crystallizability of PLA strongly depends on its enantiomeric composition and worsens with increasing content of repeating units of different chirality in the chain [6,7]. However, even optically pure poly(L-lactide) (PLLA), if cooled sufficiently fast, remains amorphous and vitrifies. Then, upon heating from the glassy state, such amorphous PLLA can cold-crystallize. However, not only the enantiomeric composition but also other factors, including molar mass and macromolecular architecture, for instance, branching or star structure, are also important and influence the crystallization of PLA. Depending on crystallization temperature, PLA chains crystallize from melt in the ordered alpha or disordered alpha’ orthorhombic forms [8], which can be identified not only by wide angle X-ray scattering but also by Raman spectroscopy or nuclear magnetic resonance spectroscopy [9,10].

Star polymers are especially interesting for many applications, for example in biomedicine or engineering [11,12]. Their rheological, thermal and mechanical properties can differ from those of their linear counterparts [11,13]. In addition, processing of star polymers can be carried out at lower temperatures than their linear analogs, which could be beneficial, especially in the case of polymers prone to thermal degradation like PLAs.

Star PLLAs were most often obtained by the bulk polymerization of cyclic lactide conducted at a temperature above the melting point of the monomer (∼99 °C), in the presence of initiators with hydroxyl end groups, with stannous octoate (Sn(Oct)_2_) as a catalyst [14,15,16,17,18,19]. Crystallization of star PLLAs was found to be affected by their molar masses and numbers of arms. Usually, PLLAs with number average molar masses, M_n_, below 100 kg/mol were analyzed. The consequences of the relatively small molar mass of star PLLA are short arm length resulting in a small number or even absence of entanglements and a large number of chain ends as well as branching points. The presence of branching points, initiator moieties, especially bulky ones, in the middle of macromolecules, hydroxyl chain end groups enhancing hydrogen bonding, and chain directional change at the branching points, are the factors disturbing the segmental mobility [11,20]. Moreover, the branching points have to be excluded from the crystalline regions [21]. For example, for PLLAs with M_n_ about 35 kg/mol, nonisothermal crystallization peak temperature, T_c_, and overall isothermal crystallization rate decreased upon the increasing number of arms up to six [22]. Moreover, an increase of T_g_, cold crystallization peak temperature, T_cc_, accompanied by a decrease of melting peak temperature, T_m_, and crystal growth rate of 3-arm PLLAs, with M_n_ in the range of 13 to 63 kg/mol, were observed, as compared with those of linear PLLAs [21]. It is worth noting that crystallization of 6-arm PLLAs was rarely examined, and the studies were limited to polymers with M_n_ per one arm below 10 kg/mol. For instance, it was found [23] that for 1-, 2-, 4- and 6-arm PLLAs, T_m_, T_cc_, and crystallinity, χ_c_, decreased with increasing number of arms at a fixed M_n_. Recently, Bojda et al. [24] synthesized three star PLLAs, two 6-arm with weight average molar masses, M_w_, of 120 and 245 kg/mol and one 4-arm with M_w_ of 123 kg/mol, and compared their crystallization with that of linear ones with M_w_ of 121, 291 and 339 kg/mol. At M_w_ close to 120 kg/mol the star architecture decreased the crystal growth rate in the temperature range of 120–145 °C. Crystal growth of PLLAs with M_w_ > 200 kg/mol was the slowest and unaffected by the macromolecular architecture. The slow crystal growth in these PLLAs resulted in their weak crystallization during cooling.

In turn, it is long known that the crystallization of polymers, including PLAs, is strongly influenced by flow, which plays a vital role during industrial processing. The flow-induced macromolecular orientation can strongly affect the crystallization kinetics and the resulting structure, which are controlled by the interplay between crystallization and chain relaxation. The fundamental processes governing the flow-induced crystallization of polymer melts were discussed by many authors [25,26,27]. The shear-induced crystallization strongly depends on the temperature of shearing, T_s_, shear rate, $\dot{\gamma }$, and total strain. It is believed [28] that to induce the point-like nuclei and fibrillar nuclei, the shear rate has to exceed the inverse reptation time and the inverse Rouse relaxation time of the high molar mass tail, respectively, although when the flow is strong enough, but too short, intermediate regimes were also defined. It is worth noting that others postulated that mechanical work is a controlling parameter [26,29,30]. A very important factor is the polymer molecular characteristic, especially the high molar mass tail is crucial due to the vital role of the longest macromolecules in the flow-induced crystallization. [26,31,32]. Similarly to other polymers, the enhancement of point-like nucleation, formation of oriented nuclei and shish-kebab structures induced by shear were observed in PLAs [33,34,35]. The effect of shear on both isothermal [34,35,36,37] and nonisothermal crystallization of PLAs was studied. Bojda et al. [36] demonstrated that smaller content of D-lactide enhanced the effect of shear on nonisothermal crystallization on PLA and that higher crystallinity degree developed during slower post-shearing cooling. In turn, Kim et al. [38] compared shear-induced crystallization of linear and 4-arm PLLAs with M_w_ of about 2 kg/mol and found that that of the latter was slightly faster.

To the best of our knowledge, the effect of shear flow on the crystallization of star PLLAs with higher molar masses was not investigated. Only shear-induced isothermal crystallization of PLAs with long chain branching (LCB), prepared by γ irradiation, was studied in [39,40]. It was demonstrated that the shear-induced nucleation density in LCB PLA was strongly enhanced in comparison to linear PLA and increased with increasing LCB degree. Moreover, the transformation from spherulitic to oriented crystalline morphologies was observed. It was concluded that the shear-induced formation of the oriented crystalline morphology of LCB PLAs was related to the hindering of relaxation of the stretched LCB macromolecular chain network.

In the present study, shear-induced nonisothermal crystallization of star PLLAs with M_w_ close to 120 and 240 kg/mol was studied and compared with that of their linear analogs. In addition, the crystallization of linear PLLA with M_w_ of 339 kg/mol was also examined. The polymers were sheared at 170 and 150 °C and cooled at 10 or 30 °C/min. The crystallization was followed by a light depolarization technique, whereas the crystallized specimens were ex-situ examined with scanning electron microscopy (SEM), differential scanning calorimetry (DSC), small- and wide-angle X-ray scattering (2D-SAXS and 2D-WAXS).

## 2. Materials and Methods

Linear PLLAs having at one chain end benzyl alcohol and star-shaped PLLAs having as a core: di(trimethylolpropane) (4-arm PLLA-OH) or dipentaerythritol (6-arm PLA-OH), were synthesized in bulk at 130 °C by coordination polymerization using hydroxyl compound as an initiator and stannous octoate as a catalyst, as described previously [24]. The resulting PLLAs were dissolved in dichloromethane and precipitated into methanol, separated by filtration, and washed several times with methanol. The polymers were characterized with size exclusion chromatography (SEC) and ^1^H NMR. After purification, the polymers were stabilized with 0.2 wt.% of Irganox 1010 and 0.2 wt.% of Irganox 1024, both from BASF (Ludwigshafen, Germany). The details of polymerization, purification, characterization and stabilization were previously described [19,24,41,42].

The average molar masses, number, M_n_, weight, M_w_, and z-average, M_z_, and dispersity of linear and star-shaped PLLAs measured by SEC in dichloromethane are given in Table 1. 

For studies of crystallization, 200 μm thick films were compression moulded at 200 °C for 3 min in a hydraulic press and quenched to room temperature, RT, between metal blocks. 

Flow-induced crystallization was carried out in the Linkam CSS-450 optical shearing system (Linkam, Waterfield, UK) mounted in a polarizing light microscope (PLM) Nikon Eclipse 80i equipped with Nikon DS Fi1 video camera. The films were: heated to 210 °C at 30 °C/min and held at 210 °C for 3 min to erase the thermal history. Next the films were cooled at 30 °C/min to T_s_ of 170 or 150 °C and sheared at a rate, $\dot{\gamma }$, of 5, 10 and 20/s for 20, 10 and 5 s, respectively to reach the same strain of 100. After shearing, they were cooled to RT at a rate, v, of 10 or 30 °C/min. The shearing conditions were selected based on preliminary studies. Control specimens were subjected to a similar thermal treatment in quiescent conditions; they were held at T_s_ for 20 s without shearing. 

The conversion of melt into the crystalline phase was followed using the light depolarization method. The intensity of transmitted depolarized light was measured during cooling and the relative volume conversion degree, α_vr_(T), was calculated according to the expression:

α_vr_(T) = [I(T) − I(T_o_)]/[I(T_e_) − I(T_o_)] (1)

where: I(T) denotes the intensity of transmitted depolarized light at temperature T, whereas T_o_ and T_e_ are the initial temperature and the final temperature of the measurement. 

In the plate-plate geometry, shear rate varies along a radius, hence for ex-situ examination, specimens were cut from the films at proper distances from the centers, at which shear rates were equal to the selected values.

Crystallinity and thermal properties of the specimens were analyzed with differential scanning calorimetry (DSC) using TA Instrument DSC TA Q20 (New Castle, USA) during heating at 5 °C/min from RT, under nitrogen flow.

Crystal orientation in the films was examined with 2D-WAXS in the transmission mode, using a WAXS camera coupled to an X-ray generator (sealed-tube, fine point CuKα source, Ni filtered, operating at 50 kV and 35 mA) from Philips (Eindhoven, Netherlands). The incident beam was normal to the film plane. The lamellar structure was probed with 2D small angle X-ray scattering (2D-SAXS). Kiessig-type camera with the sample-detector distance of 1.2 m was coupled to GeniX Cu-LD X-ray system from Xenocs (Grenoble, France), with CuKα source operating at 50 kV and 1 mA. The incident beam was normal to the film plane. The scattering patterns were recorded with Pilatus 100K solid-state detector from Dectris (Baden, Switzerland). 

To reveal their internal structure, selected sheared PLLA specimens were cut across their thickness parallel to the shearing direction, and the exposed surfaces were analyzed with scanning electron microscopy (SEM) using Jeol JSM-5500 LV (Tokyo, Japan). Before the examination, the specimens were etched according to the known method [36,43], at 37 °C, in a solution of 61 mg of Trizma base, 2 mg of sodium azide and 4 mg of Proteinase K (all from Sigma-Aldrich, St. Louis, MO, USA) in 5 mL of distilled water. After appropriate washing and drying, the specimens were sputtered with gold. 

## 3. Results and Discussion

### 3.1. Crystallization

**Figure 1:**
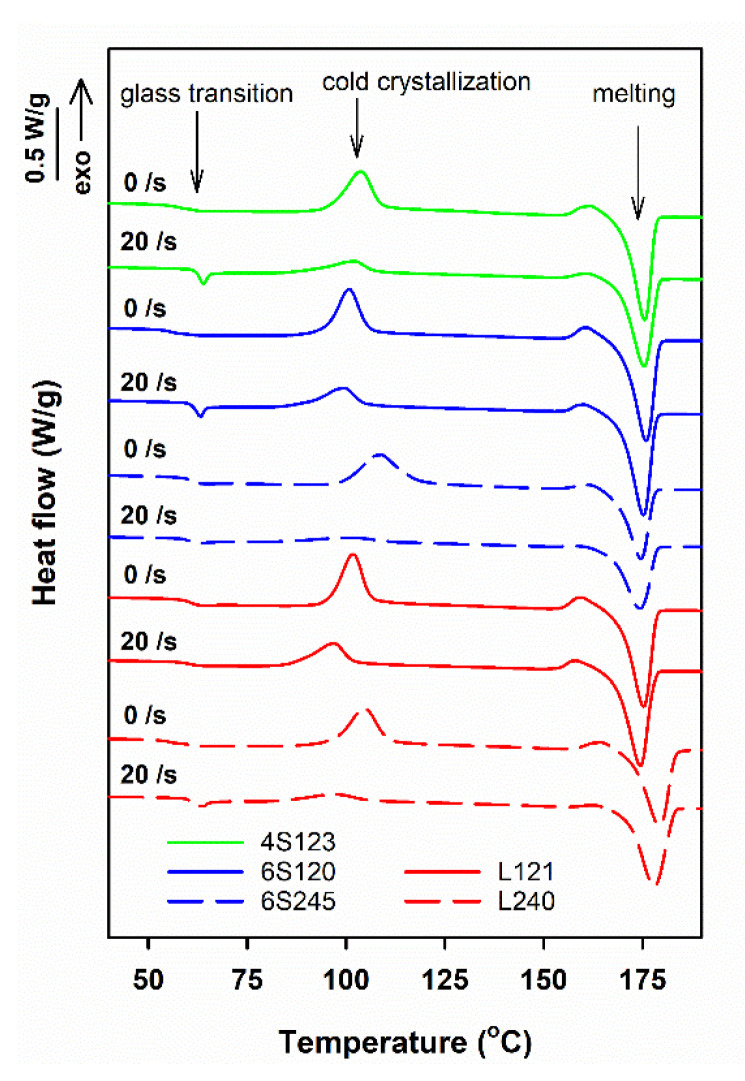
DSC heating thermograms of PLLAs sheared at 20/s for 5 s at 150 °C and next cooled at 30 °C/min, and thermograms of control specimens cooled at 30 °C/min. The curves shifted vertically for clarity.

Exemplary DSC heating thermograms of star-shaped and linear PLLAs sheared at 170 °C and then cooled to RT at 30 °C/min, collected in Figure 1, exhibit glass transition with T_g_ at approx. 60–61 °C, cold crystallization exotherms and melting endotherms, with peaks at T_cc_ close to 100 °C and T_m_ above 170 °C, respectively. In addition, on the thermograms, small pre-melting exotherms are visible, with maxima close to 160 °C, which originated from the recrystallization of the disordered alpha’ to the ordered alpha orthorhombic form [8]. During heating at 5 °C/min, the cold-crystallization occurred in a relatively low temperature range and the crystallization exotherms and the melting endotherms did not overlap, which facilitated integration of the peaks and calculation of enthalpies of the processes. The melting enthalpy, ΔH_mc_, of crystals formed during cooling, before the heating in DSC, was calculated by subtracting the enthalpies of exothermic effects of crystallization and recrystallization, ΔH_cc_ and ΔH_rc_, respectively, from the melting enthalpy, ΔH_m_. 

For the control specimens, ΔH_mc_ was small, or even close to zero, indicating that they were amorphous or with low crystallinity. However, ΔH_mc_ of the sheared specimens was markedly larger, proving that crystallinity developed in PLLAs during the post-shearing cooling. The differences between the thermograms in Figure 1 evidence that the effect of shear depended on the molar mass, but also on the macromolecular architecture of PLLAs studied. 

It must be noted that such approach does not take into account the temperature dependence of heat of fusion, due to which ΔH_cc_ of the cold-crystallized crystals can be lower than their melting enthalpy. However, although T_m_ – T_cc_ was up to about 70 °C, in most cases ΔH_cc_ was significantly smaller than ΔH_mc_, therefore reducing the overestimation of the latter. Another effect that should be considered is the difference in the heat of fusion of the ordered alpha form and the disordered alpha’ form of PLLA. It is known that between 100 and 120 °C PLLA crystallizes not only in the ordered alpha modification but also in the disordered alpha’ form. Below 100 °C, PLLA crystallizes from the quiescent melt only in the alpha’ form [8], although the alpha form was found after shear-induced crystallization at 96 °C [44]. Although the heat of fusion of the alpha’ crystals is significantly lower than that of the alpha modification [45], the influence of that on ΔH_mc_ can be neglected, because of the alpha’ to alpha recrystallization prior to melting. In addition, the alpha’ to alpha recrystallization occurring near 160 °C and also reorganization occurring in the alpha phase prior to melting, further reduce the possible overestimation of ΔH_mc_.

**Figure 2:**
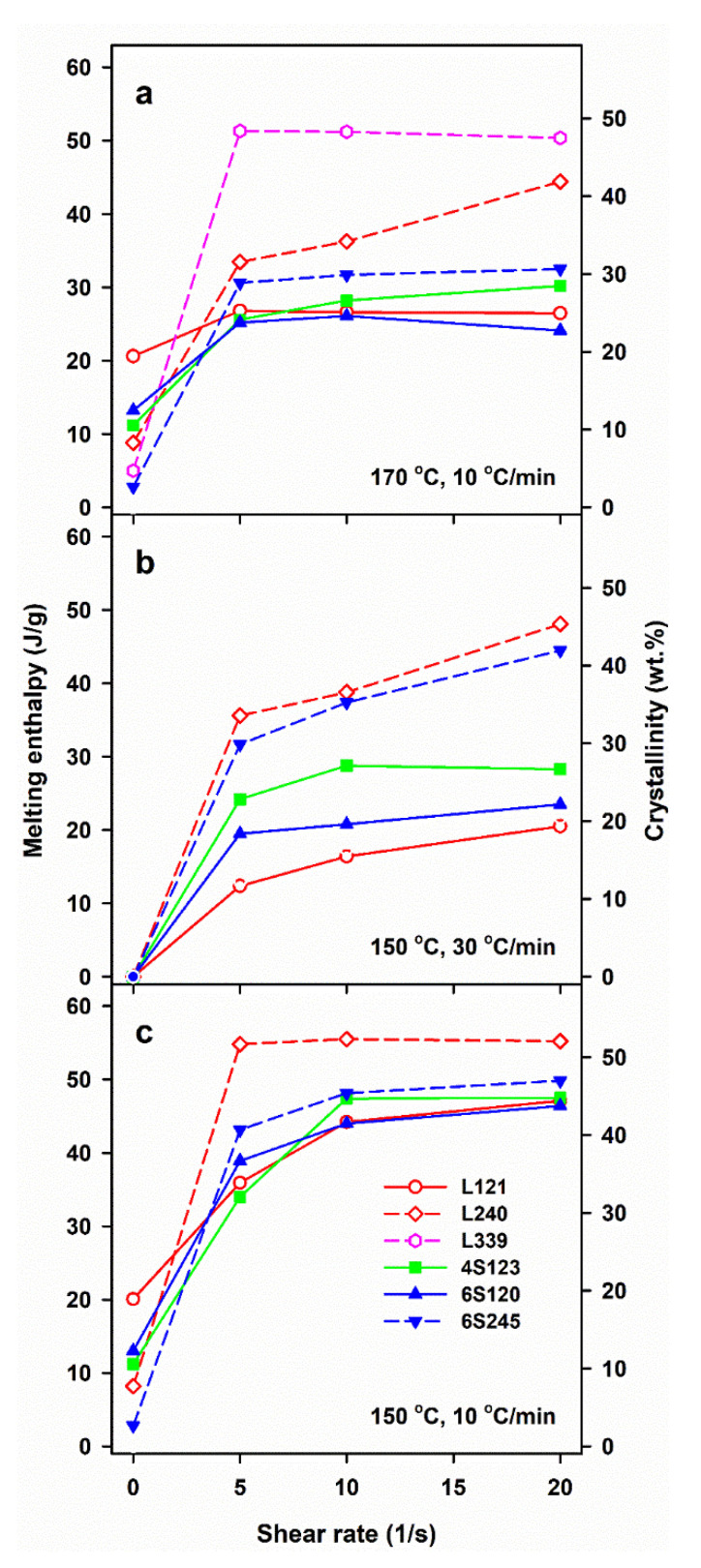
Melting enthalpy, ΔH_mc_, of crystalline phase formed in PLLAs during cooling at 10 and 30 °C/min after shearing at 170°C (**a**) and 150 °C (**b**,**c**) versus shear rate,$\;\dot{\gamma }$.

Figure 2 illustrates the effect of shearing conditions and post-shearing cooling on ΔH_mc_ and mass crystallinity, χ_c_ of linear and star PLLAs. The ΔH_mc_ values are averages, based on at least two or three measurements. The mass crystallinity, χ_c_, was calculated from ΔH_mc_, assuming that the heat of fusion of 100% crystalline PLLA is 106 J/g [46].

The control specimens cooled at 30 °C/min were practically amorphous, whereas in those cooled at 10 °C/min crystallization occurred, although ΔH_mc_ and the corresponding χ_c_ were small, being the largest for L121, close to 20 J/g and 19%, respectively, as previously found for the same polymer [24]. Shearing at 150 and 170 °C enhanced crystallization in all PLLAs. In general, ΔH_mc_ increased with $\dot{\gamma }$, although weakly in most cases, or was even independent of $\dot{\gamma }$, most possibly due to the same final shear strain achieved during all experiments. Moreover, ΔH_mc_ of L339 sheared at 170 °C and L240 sheared at 150 °C, and next cooled at 10 °C/min, reached very high values even for $\dot{\gamma }$ of only 5/s. 

Shearing at 170 °C followed by cooling at 30 °C/min resulted in rather low ΔH_mc_ of all PLLAs, up to about 15 J/g, except L240 and L339, for which ΔH_mc._ values were higher. A decrease of v to 10 °C/min enhanced the post-shearing crystallization in all PLLAs. As seen in Figure 2a, ΔH_mc_ values of PLLAs with M_w_ close to 120 kg/mol ranging from 24 to 30 J/g were similar. Slightly higher ΔH_mc_ of 31–32 J/g was found for 6S245, and even higher up to 44 J/g for L240. The effect of shear on the crystallization of L339 was the strongest, which was reflected in ΔH_mc_ close to 50 J/g. A decrease of T_s_ to 150 °C intensified the post-shearing crystallization at both cooling rates. L339 crystallized during shearing at 150 °C; hence, studies of its post-shearing nonisothermal crystallization were impossible. As seen in Figure 2b, ΔH_mc._ values of PLLAs with M_w_ close to 120 kg/mol, cooled at 30 °C/min, increased up to 21–29 J/g, and those of 6S245 and L240 to 44 and 48 J/g, respectively. Slower cooling, at 10 °C/min, resulted in higher ΔH_mc_, in the range of 34–47 J/g for PLLAs with M_w_ close to 120 J/g, whereas in the range of 43–49 J/g and 55 J/g for 6S245 and L240, respectively, as s shown in Figure 2c.

**Figure 3:**
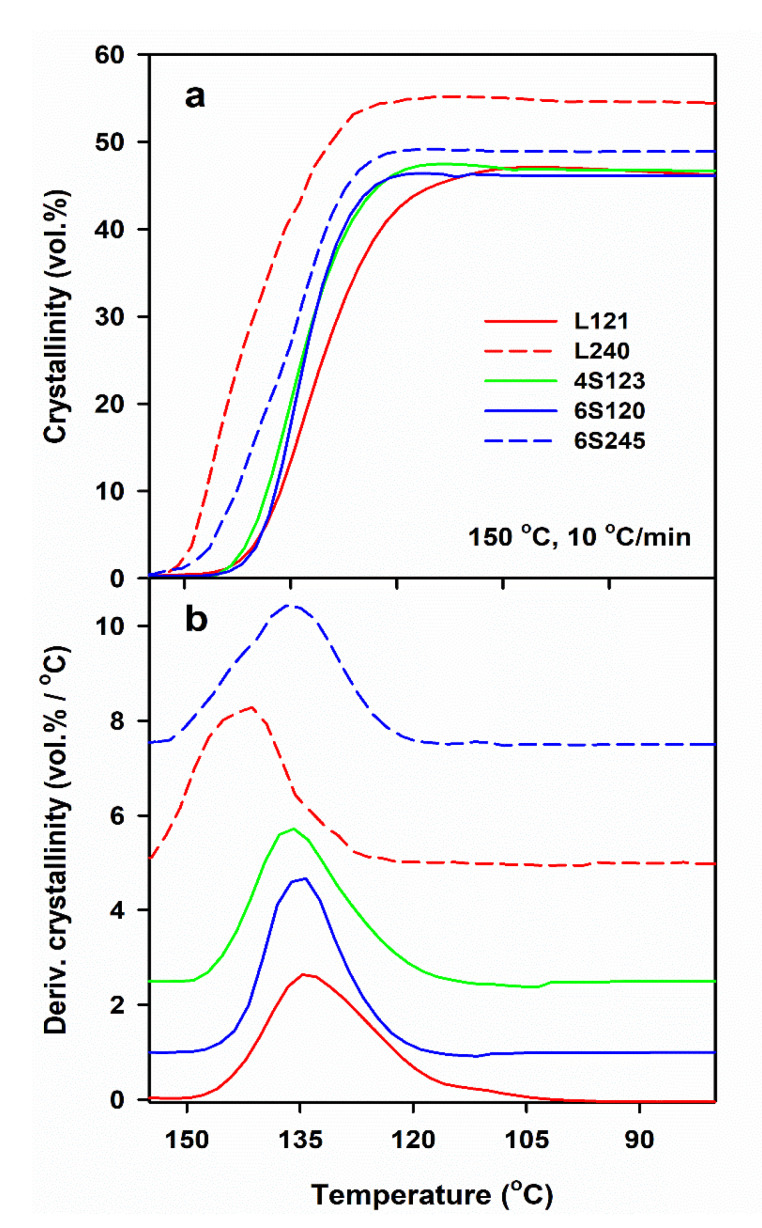
Development of crystallinity determined by light depolarization method (**a**) and derivative of crystallinity with respect to temperature (**b**) in PLLAs during cooling at 10 °C/min after shearing at 20/s, 150 °C. The curves in Figure 3b shifted vertically for clarity.

**Figure 4:**
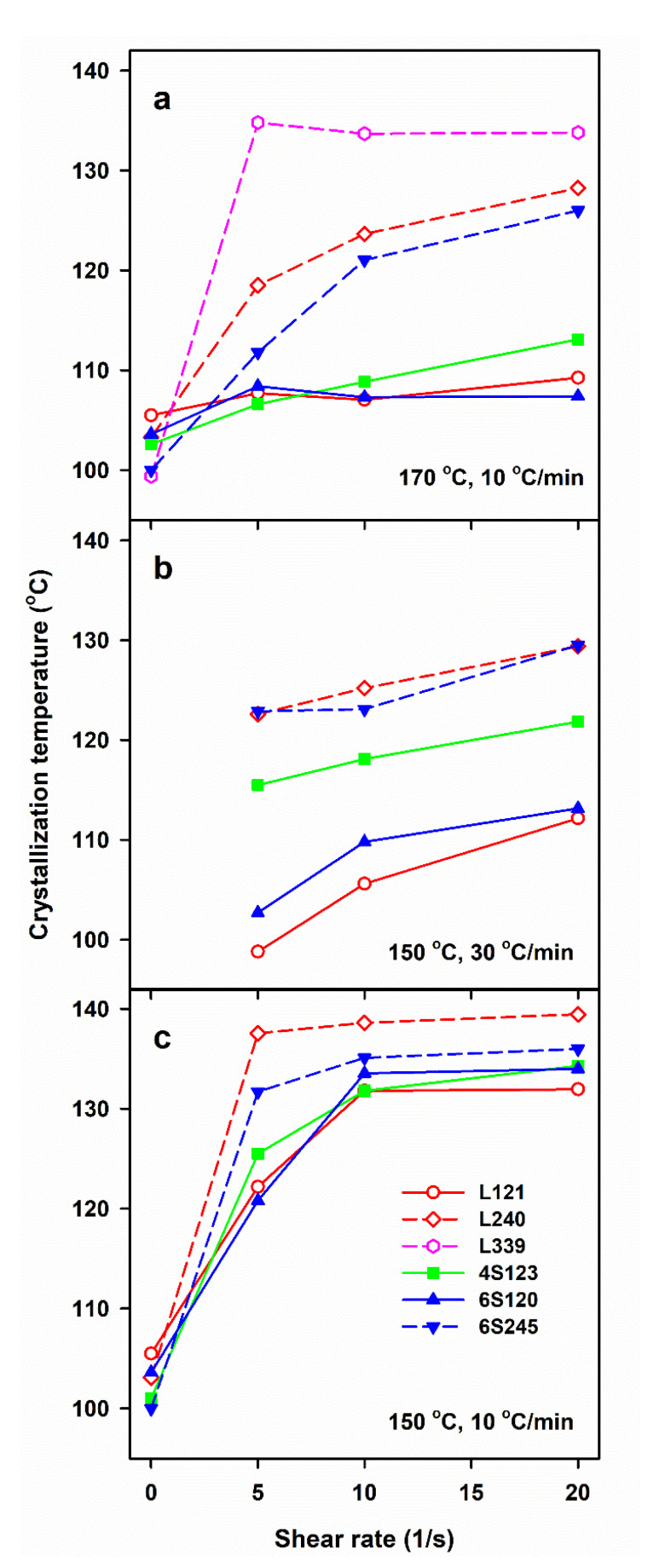
Crystallization peak temperature, T_c_, during cooling of PLLAs at 10 and 30 °C/min after shearing at 170 °C (**a**) and 150 °C (**b**,**c**) versus shear rate, $\dot{\gamma }$.

DSC measurements allow us to determine only the final χ_c_ developed during post-shearing cooling, whereas the light depolarization method enables us to follow the increase of α_vr_ during crystallization. To compare the crystallinity increase in specimens with different final crystallinity, volume crystallinity α_v_(T) equal to α_vr_(T) χ_v_, was plotted in Figure 3, where χ_v_ is the final volume crystallinity calculated based on χ_c_ and the densities of the amorphous and crystalline phases of PLA [47]. As it is explained above, χ_c_ was calculated based on the melting enthalpy of crystals formed during post-shearing cooling, ΔH_mc_. It should be mentioned that the lower melting enthalpy of the alpha’ phase was not accounted for because ΔH_mc_ was determined from the melting endotherm preceded by the pre-melting recrystallization of alpha’ to alpha form. Differentiation of α_v_(T) with respect to temperature permitted to obtain the temperature dependencies of crystallization rate. It appears that the lower T_s_, slower cooling and higher M_w_ of PLLA resulted in the higher temperature range of crystallization. The effect of $\dot{\gamma }$, T_s_ and v, as well as of M_w_ of PLLA and its macromolecular architecture, on T_c_ was similar to that on ΔH_mc_, as it is shown in Figure 4. T_c_ correlated with ΔH_mc_ and crystallinity, the higher the former the larger the latter. T_c_ increased with decreasing T_s_ and v, and with increasing $\dot{\gamma }$. The highest T_c_ values were found for L339, lower for L240 and 6S245, and even lower for 4S123, 6S120 and L121, showing the influence of M_w_, but also of the macromolecular architecture.

**Figure 5:**
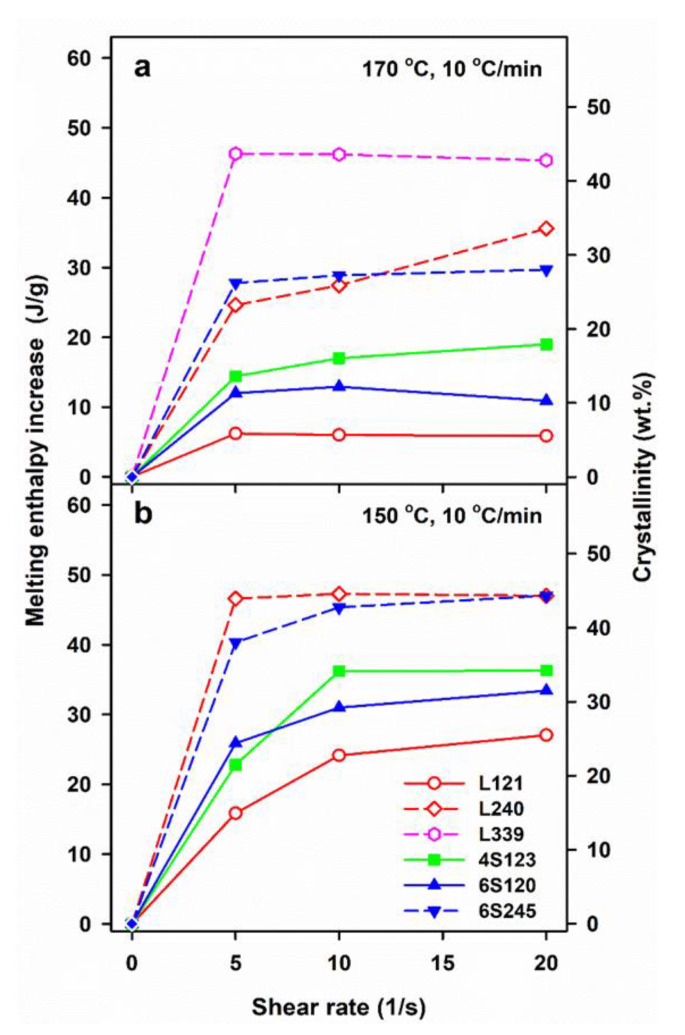
Increase of melting enthalpy, ΔH_mc_-ΔH_mc_^q^, of crystalline phase formed in PLLAs during cooling at 10 °C/min caused by shearing at 170 °C (**a**) and 150 °C (**b**) versus shear rate, $\dot{\gamma }$. ΔH_mc_^q^ denotes the melting enthalpy of crystals formed during cooling at 10 °C/min in control specimens.

The results show a crucial role of T_s_ and v. The lower T_s_ increased the relaxation times of macromolecules and lowered the energy barrier for nucleation, thus enhancing the shear-induced crystallization. In turn, the slower cooling enabled a longer time for crystallization before too low temperature was reached, increasing therefore T_c_, ΔH_mc_ and χ_c_, the latter determined based on ΔH_mc_. However, not only the shearing conditions and v determined the post-shearing crystallization. T_c_ and ΔH_mc_ were strongly influenced by molar masses of PLLAs, as can be expected, but they were also affected by macromolecular architecture. Figure 2b shows that in the case of cooling at 30 °C/min, the shearing at 150 °C had the weakest effect on L121, stronger on 6S120, and the strongest on 4S123 crystallization. Figure 2a,c show that during cooling at 10 °C/min T_c_ and ΔH_mc_ values of all PLLAs with M_w_ near 120 kg/mol were similar. However, it must be reminded that in the temperature range of 120–145 °C the crystal growth rate of L121 was higher than that of the other PLLAs studied [24]. The crystallization kinetics is governed by both the crystal growth rate and the nucleation rate [48], hence, similar T_c_ and ΔH_mc_ values of L121, 6S120 and 4S123 are suggestive of much stronger nucleation in the two latter. The control specimens cooled at 30 °C/min were practically amorphous but crystallized during cooling at 10 °C/min. The shear-induced increase of ΔH_mc_ and T_c_ of specimens cooled at 10 °C/min is plotted in Figure 5 and Figure S1 in Supplementary Information (SI), respectively. The plots clearly show that the shear enhanced more the crystallization of 4S123 and 6S120 than that of L121, despite the higher M_z_ of the latter.

The enhancement of the effect of shear in 6S120 and 4S123 as compared to L121 was undoubtedly caused by the star architecture of macromolecules, which hindered the relaxation of the stretched macromolecular chain network. In contrast to that, the shear-induced crystallization in 6S245 and L240, was similar, and even stronger in the latter, as shown in Figure 2 and Figure 5. Although 6S245 and L240 had similar M_n_ and M_w_, M_z_ of L240, 414 kg/mol, exceeded that of 6S245, 294 kg/mol, evidencing the higher content of larger macromolecules, which presence compensated the effect of 6S245 star architecture on the shear-induced crystallization. In the flow-induced crystallization of a polymer a high molar mass tail of its molar mass distribution plays a crucial role, due to long relaxation times, and at M_w_ of 240–245 kg/mol its effect compensated that of star architecture. It is also of importance that due to its higher molar mass, the number of branching points in 6S245 was smaller than in 6S120, hence their effect on the macromolecular mobility was reduced.

**Figure 6:**
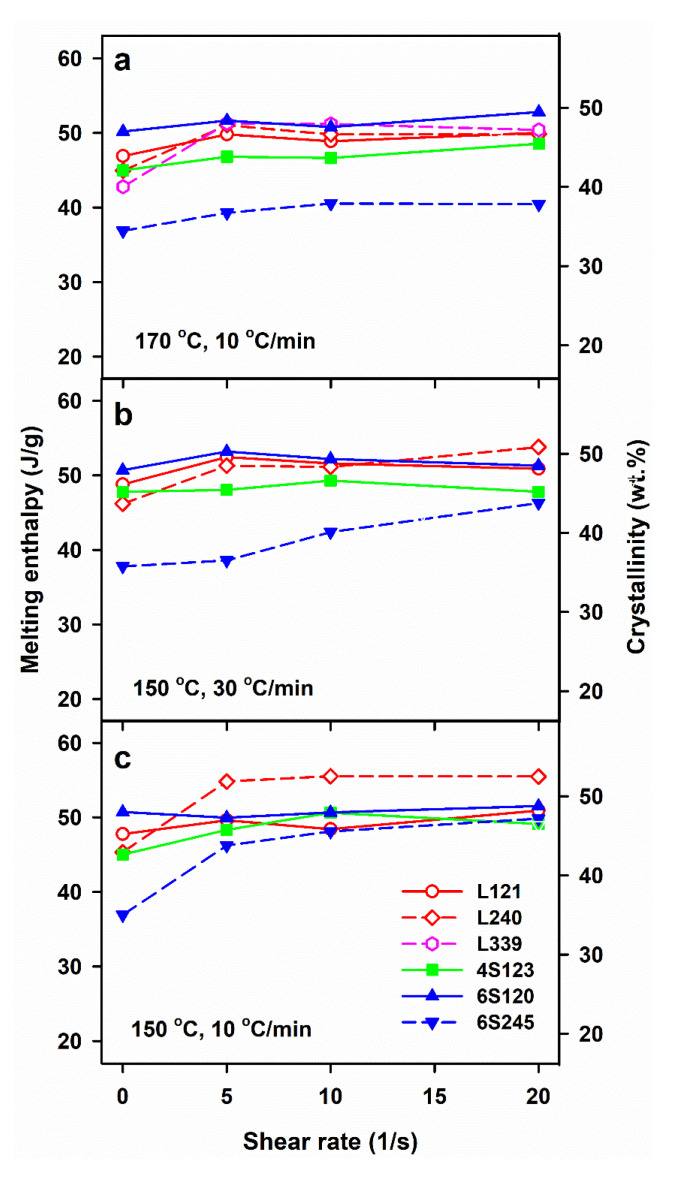
Melting enthalpy, ΔH_m_, measured during DSC heating at 5 °C/min of PLLAs previously cooled at 10 and 30 °C/min, after shearing at 170 °C (**a**) and 150 °C (**b**,**c**) versus shear rate, $\dot{\gamma }$.

The crystallization, which was not completed during cooling continued during subsequent heating in DSC, resulting in cold-crystallization exotherms with peaks at T_cc_ of 97–109 °C, as shown in Figure 1. In many cases, pre-melting exotherms, with maxima at 159–164 °C, evidenced the alpha’ to alpha form recrystallization. Usually, single melting peaks were observed, with T_m_ of 174–179 °C, although some of them with shoulders. As shown in Figure 6, ΔH_m_ values of the control specimens of 6S245 were equal to 37–38 J/g, whereas those of the other control PLLAs studied were higher, ranging from 43 to 51 J/g. The sheared PLLAs exhibited increased ΔH_m_ of 39–55 J/g. The lowest values of ΔH_m_ were those of 6S245 cooled at 10 °C/min after shearing at 170 °C, and cooled at 30 °C/min after shearing at 150 °C, as seen in Figure 6. In turn, among PLLAs sheared at 150 °C and cooled at 10 °C/min, L240 exhibited the highest ΔH_m_, as evidenced in Figure 6c. These differences reflect the different ability of PLLAs studied to crystallize, as described in [24], and the different effect of shear influenced by the molar masses and macromolecular architecture of the studied PLLAs. 

### 3.2. Structure

**Figure 7:**
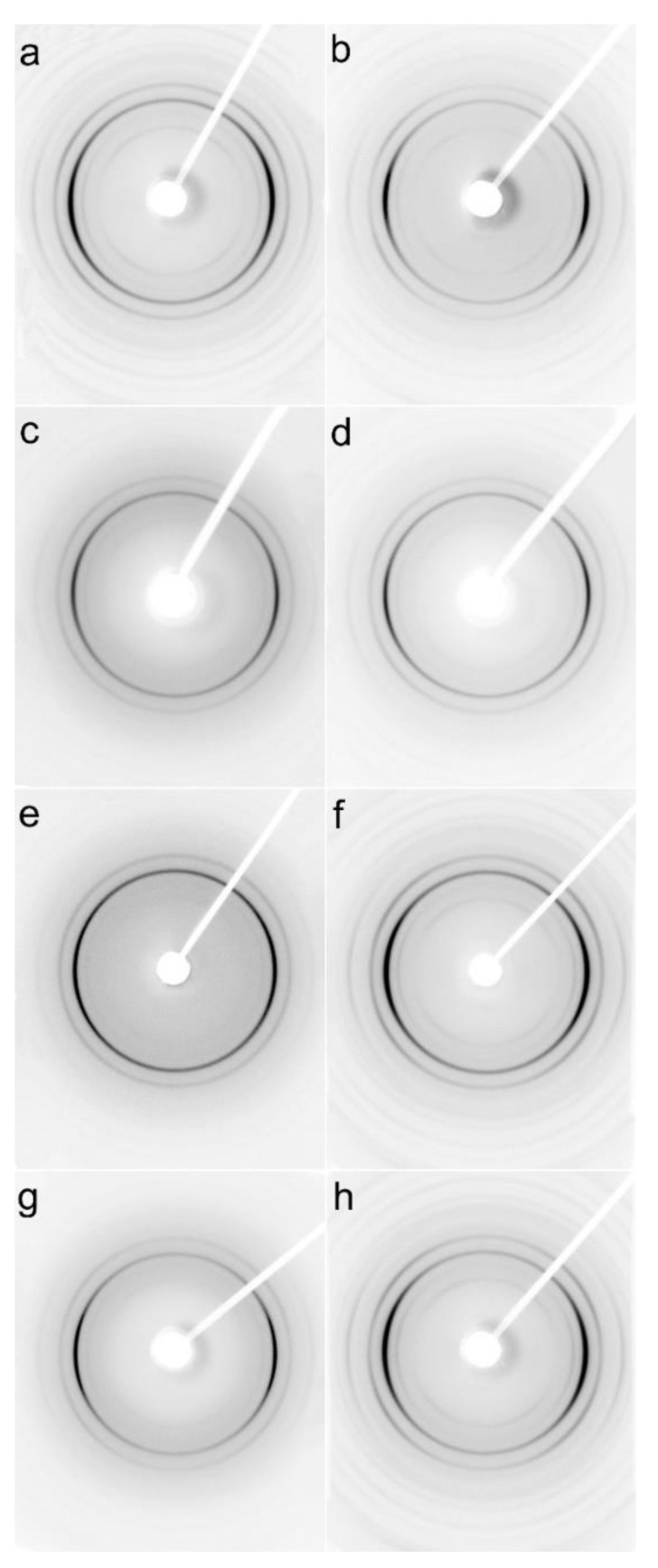
2D-WAXS patterns of PLLAs: L339 sheared at 170 °C at 5/s for 20 s (**a**) and at 20/s for 5 s (**b**) and cooled at 10 °C/min, 4S123 sheared at 150 °C at 10/s for 10 s (**c**) and at 20/s for 5 s (**d**) cooled at 30 °C/min, L240 sheared at 150 °C at 10/s for 10 s (**e**) and at 20/s for 5 s (**f**) cooled at 30 °C/min, 6S245 sheared at 150 °C at 10/s for 10 s (**g**) and at 20/s for 5 s (**h**) cooled at 30 °C/min. Shearing direction -vertical.

**Figure 8:**
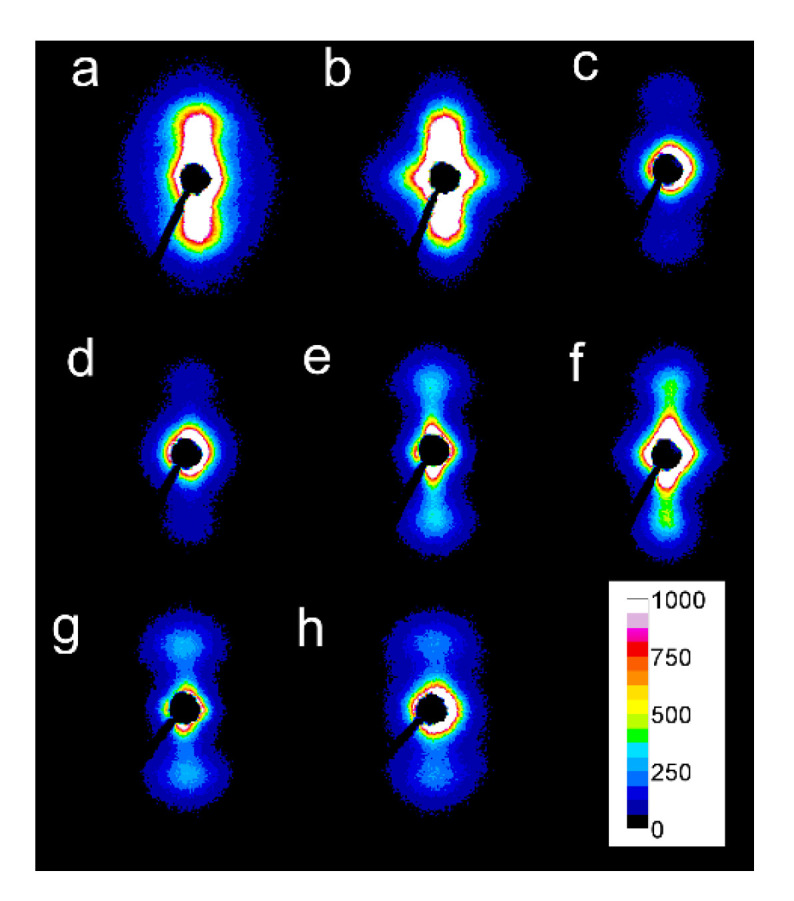
2D-SAXS patterns of PLLAs: L339 sheared at 170 °C at 5/s for 20 s (**a**) and at 20/s for 5 s (**b**) and cooled at 10 °C/min, 4S123 sheared at 150 °C at 10/s for 10 s (**c**) and at 20/s for 5 s (**d**) cooled at 30 °C/min, L240 sheared at 150 °C at 10/s for 10 s (**e**) and at 20/s for 5 s (**f**) cooled at 30 °C/min, 6S245 sheared at 150 °C at 10/s for 10 s (**g**) and at 20/s for 5 s (**h**) cooled at 30 °C/min. Shearing direction-vertical.

Examples of 2D-WAXS and 2D-SAXS patterns of sheared PLLA specimens are collected in Figure 7 and Figure 8. Generally, the intensities of the reflections from the crystalline phase correlated with χ_c_ determined from DSC thermograms and plotted in Figure 2, and increased with increasing χ_c_. (200)/(110) and (203) reflections (indicated by arrows in Figure S2 in SI) typical of both alpha and alpha’ modifications were well visible on all patterns. Also, (210) reflection near 2θ of 22° characteristic of the alpha form was present in all the patterns, evidencing that the alpha phase was formed in all sheared specimens. 

This is in accordance with the T_c_ values shown in Figure 4, which indicate that the crystallization of most of the specimens occurred fully or partially above 110 °C; the higher the T_c_ the more intense was the (210) reflection. Only very weak (210) reflections were discernible in the patterns of PLLAs (not shown), which T_c_ was close to 100 °C, for instance, L121 and 6S120 sheared at 150 °C at 5/s and cooled at 30 °C/min, which indicated small alpha content. 

In some of the 2D-WAXS patterns, intensities of (200)/(110) and (210) reflections were enhanced in equatorial regions, indicating the orientation of the respective crystallographic planes parallel to the shearing direction, and thus evidencing the orientation of polymer chain axes in the flow direction. This was corroborated by the strong polar reflections in the corresponding 2D-SAXS patterns reflecting the orientation of lamellae stacks perpendicular to the flow direction.

**Figure 9:**
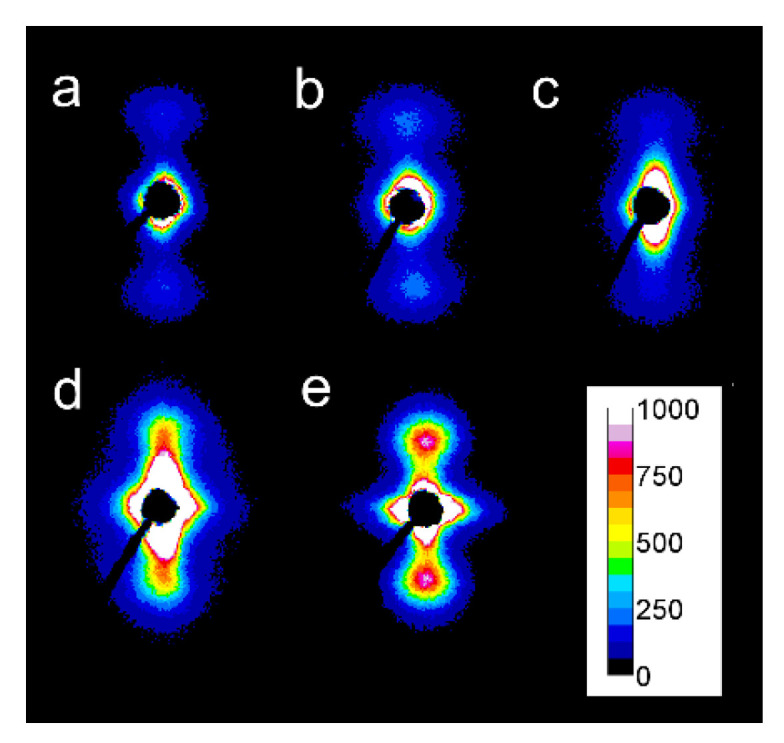
2D-SAXS patterns of PLLAs: sheared at 150 °C at 20/s for 5 s and cooled at 10 °C/min: L121 (**a**), 4S123 (**b**), 6S120 (**c**), L240 (**d**), 6S245 (**e**). Shearing direction–vertical.

Among the specimens sheared at 170 °C and next cooled at 10 °C/min, L339 clearly exhibited such orientation, as shown in Figure 7a,b, and in Figure 8a,b. Weaker orientation was also detected in 6S245 and L240 sheared at 20/s (not shown). Shearing at 150 °C followed by cooling at 30 °C/min resulted in the crystal orientation in L240, 6S245, and 4S123 evidenced in Figure 7c–g and Figure 8c–g. The orientation, although weaker, was also reflected in the patterns of L121 sheared at 20/s, 6S120 sheared at 10/s and 20/s (not shown). 2D-WAXS and 2D-SAXS patterns of PLLAs sheared at 150 °C and next cooled at 10 °C/min evidenced the same features of the morphology. It is well visible in 2D-SAXS patterns collected in Figure 9a–e, in which scattering from the crystalline phase was enhanced due to the higher χ_c_ developed during slower cooling.

**Figure 10:**
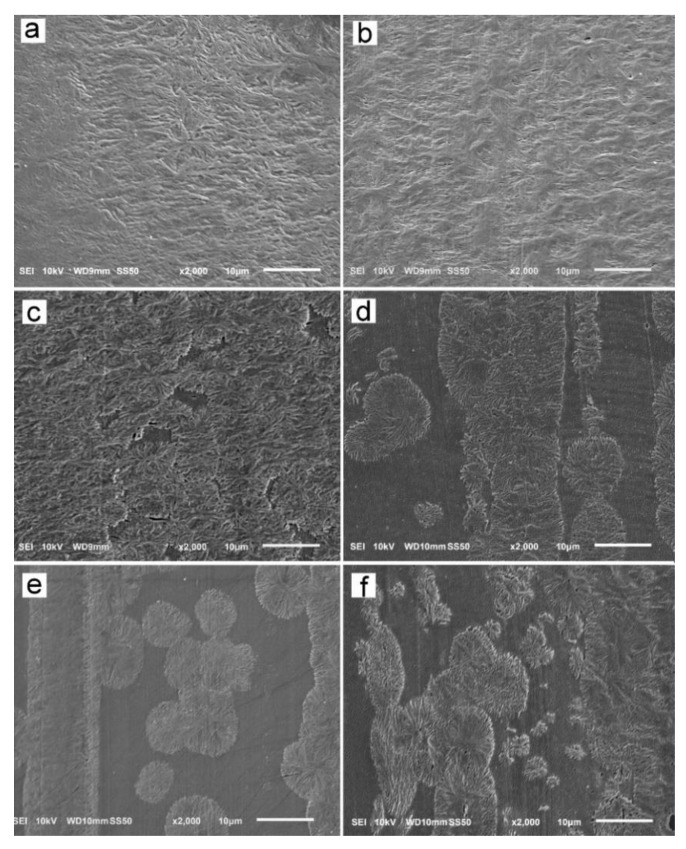
SEM micrographs of etched cross-section surfaces of PLLAs: L339 sheared at 170 °C at 5/s for 20 s, and next cooled at 10 °C/min (**a**), and L240 (**b**) 6S245 (**c**), L121 (**d**), 4S123 (**e**), 6S120 (**f**) sheared at 150 °C at 20/s for 5 s, and next cooled at 30 °C/min. Shearing direction–vertical.

**Figure 11:**
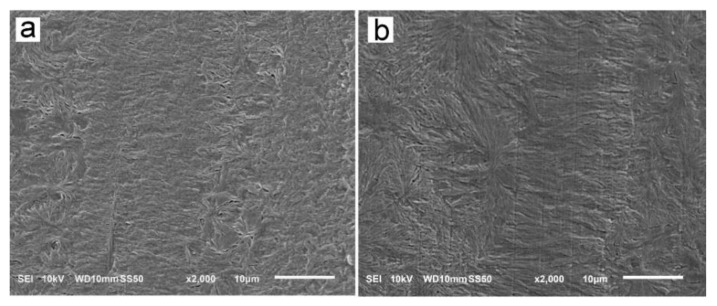
SEM micrographs of etched cross-section surfaces of L240 (**a**) and L121(**b**) sheared at 150 °C at 20/s for 5 s, and next cooled at 10 °C/min. Shearing direction—vertical.

The reason for the orientation of crystals was the shear-induced formation of fibrillar nuclei aligned in the shearing direction on which grew lamella stacks perpendicular to the shearing direction. This is well seen in SEM micrographs of cross-section surfaces of sheared PLLAs, presented in Figure 10. The micrograph of L339 sheared at 170 °C at 20/s and next cooled at 10 °C/min, in Figure 9a, evidences the presence of lamellar stacks perpendicular or nearly perpendicular to the shearing direction, and lamella fans developed from the stacks. The stacks form cylindrical structures suggestive of nucleation on fibrillar nuclei. Similar morphology was found in L240 sheared at 150 °C at 20/s, and next cooled at 30 °C/min, as shown in Figure 10b, although spherulites between cylindrical structures were also discernible. Figure 10c presents 6S245 sheared at the same conditions and cooled at the same rate. In the micrograph, cylindrical structures are seen accompanied by spherulites, with radii of several micrometers. Amorphous areas are visible in few places between the spherulites, where crystallization was not accomplished. In L121, 6S120 and 4S123 sheared at 150 °C at 20/s crystallization during cooling at 30 °C/min was even less advanced, as shown in Figure 10d–f. The cylindrical structures and spherulites between them are well distinguishable, the latter more numerous than in PLLAs with higher M_w_. The effect of slower cooling on PLLAs sheared at 150 °C at 20/s is illustrated in Figure 11. The morphology of L240 and 6S245 cooled at 10 °C/min was similar to that observed after faster cooing, as exemplified in Figure 11a showing L240. In PLLAs with M_w_ close to 120 kg/mol amorphous areas were not visible any longer because of high χ_c_ reached in these polymers during slower cooling. The specimens were completely filled with cylindrical structures and spherulites between them, as seen in Figure 11b showing L121. 

2D-WAXS results evidenced orientation of the orthorhombic crystals of PLLAs with (200)/(110) and (210) crystallographic planes parallel to the shearing direction; hence, the orientation of polymer chain axes in the flow direction. It was accompanied by strong polar reflections in the corresponding 2D-SAXS patterns reflecting the orientation of lamellae stacks perpendicular to the flow direction. The orientation of crystals was stronger in PLLAs with M_w_ above 200 kg/mol than in those with M_w_ close to 120 kg/mol. However, among the latter, 4S123 exhibited the strongest orientation, whereas that of L121 was the weakest.

SEM analysis demonstrated the presence of lamellae stacks perpendicular to the shearing direction forming cylindrical structures, nucleated on fibrillar nuclei. These structures were accompanied by spherulites, especially in PLLAs with M_w_ close to 120 kg/mol. Obviously, the shear-induced fibrillar nucleation, although occurred, was less intense in these polymers than in PLLAs with higher M_w_, which corroborated the conclusions drawn based on the results of X-ray scattering experiments. 

In general, the strongest orientation of crystals was observed for the specimens, which crystallized at the highest temperatures and reached the highest χ_c_. The crystal orientation resulted from the crystal growth on the shear-induced fibrillar nuclei; the more intense the nucleation, the higher T_c_ and χ_c_. On the contrary, the weak or absent crystal orientation indicated the predominant point-like nucleation. 

## 4. Conclusions

Three star PLLAs, 6-arm with M_w_ of 120 and 245 kg/mol, 4-arm with M_w_ of 123 kg/mol, and three linear PLLAs with M_w_ of 121, 240 and 339 kg/mol, were synthesized and their shear-induced crystallization was examined. The polymers were sheared at 170 °C and 150 °C at 5/s, 10/s and 20/s for 20 s, 10 s, and 5 s, respectively, and next cooled at 10 or 30 °C/min. The shear flow induced crystallization of the PLLAs during cooling at 30 °C/min and enhanced the crystallization at 10 °C/min, which was reflected in an increase of crystallization peak temperature, T_c_, and crystallinity, χ_c_. The flow-induced orientation of crystals was evidenced by 2D-WAXS and 2D-SAXS, although dependent on shearing conditions and molecular characteristics of the polymers. The lamellae stack perpendicular to the flow direction, suggestive of fibrillar nucleation, were observed by SEM, although spherulites were found between them, especially in PLLAs with M_w_ close to 120 kg/mol. 

The results showed the crucial role of shearing temperature, T_s_, and cooling rate, v, as the lower T_s_ increases the relaxation times of macromolecules and loweres the energy barrier for nucleation, whereas the slower cooling enabled a longer time for crystallization before too low temperature was reached. The shear-induced crystallization was also strongly influenced by molar mass of PLLA, as can be expected, but it was also affected by macromolecular architecture. It was well reflected in χ_c_ of PLLAs with M_w_ close to 120 kg/mol sheared at 150 °C and cooled at 30 °C/min. The effect of shear was the weakest on L121, stronger on 6S120, and the strongest on 4S123,despite the higher M_z_ of L121. During cooling at 10 °C/min, T_c_ and ΔH_mc_ values of all PLLAs with M_w_ near 120 kg/mol were similar. However, this evidenced the stronger shear-induced nucleation in both star PLLAs than in L121, because the crystal growth in them was slower than in L121 [24]. The effect of molecular architecture on shear-induced crystallization during cooling at 10 °C/min was clearly seen in an increase of T_c_ and ΔH_mc_ in respect to the corresponding values for the control specimens, with similar thermal history but not subjected to shearing. The stronger effect of shear on 6S120 and 4S123 as compared to that on L121 undoubtedly resulted from the star architecture of macromolecules, which hindered the relaxation of the stretched macromolecular chain network. On the contrary, the shear-induced crystallization in 6S245 and L240 was similar, and even somewhat stronger in the latter. This can be understood taking into account that in the flow-induced crystallization of polymers, a high molar mass tail of molar mass distribution plays a crucial role, due to long relaxation times. M_z_ of L240, 414 kg/mol, exceeded that of 6S245, 294 kg/mol, evidencing the higher content of larger macromolecules, which at M_w_ of 240–245 kg/mol compensated the effect of star architecture on the shear-induced crystallization. Moreover, due to its higher molar mass, the number of branching points in 6S245 was smaller than in 6S120, which reduced their effect on macromolecular mobility.

## Figures and Tables

**Table 1:** The average molar masses, number, M_n_, weight, M_w_, and z-average, M_z_, and dispersity M_w/_M_n_ of star and linear PLLAs.

Sample Code	M_w_ (kg/mol)	M_n_ (kg/mol)	M_z_ (kg/mol)	M_w_/M_n_
L121	121	81	194	1.5
L240	240	157	414	1.3
L339	339	257	495	1.3
4S123	123	97	152	1.3
6S120	120	80	162	1.5
6S245	245	183	294	1.3

## Data Availability

The data presented in this study are available on request from the corresponding author.

## Supplementary Materials

The following are available online. Figure S1: Increase of crystallization peak temperature, T_c_−T_c_^q^, of PLLAs during cooling at 10 °C/min, caused by shearing at 170 and 150 °C, versus shear rate, $\dot{\gamma }$. T_c_^q^ denotes the crystallization peak temperature of control specimens during cooling at 10 °C/min. Figure S2, 2D-WAXS pattern of PLLA L339 sheared at 170 °C at 5/s for 20 s and next cooled at 10 °C/min, with arrows indicating characteristic reflections.

## Sample Availability

Samples of the compounds are not available from the authors.
