# Supplementary material for: Shear-Induced Crystallization of Star and Linear Poly(L-lactide)s
Source: Molecules. 2021 Oct 31;26(21):6601. doi: 10.3390/molecules26216601 (PMC8588257; doi:10.3390/molecules26216601)
Supplement: Supplementary file 1 [file molecules-26-06601-s001.zip › molecules-1410341-supplementary.pdf]

Supplementary Information

# Shear induced crystallization of star and linear poly(L-lactide)s

J. Bojda*, E. Piorkowska, G. Lapienis, A.Michalski
Centre of Molecular and Macromolecular Studies, Polish Academy of Sciences,
90-363 Lodz, Poland, Sienkiewicza 112

**corresponding author :* jbojda@cbmm.lodz.pl

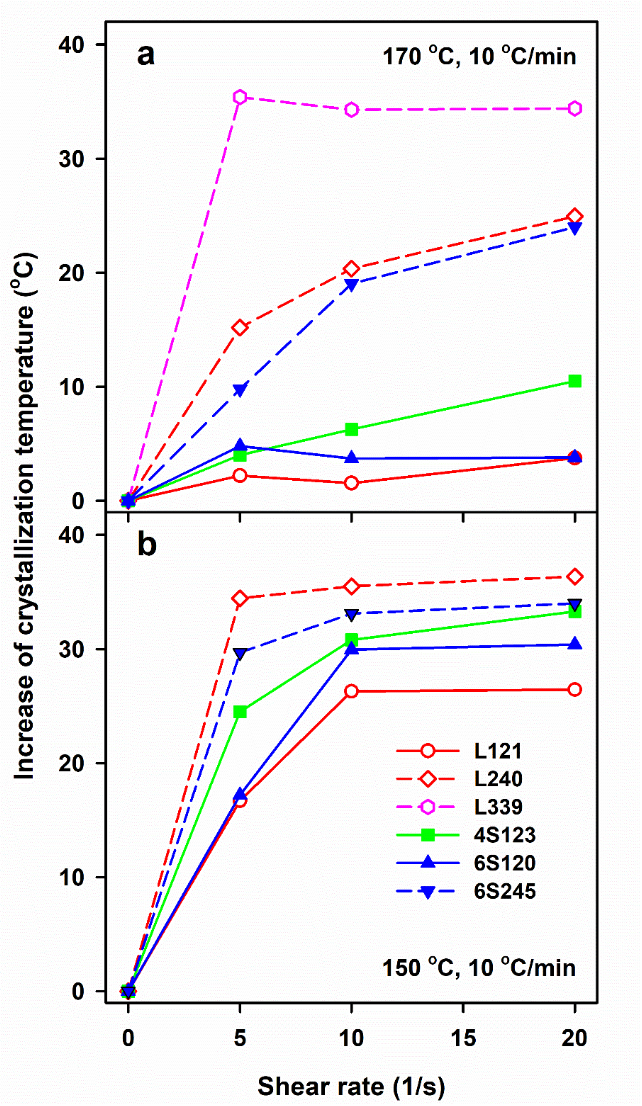


Figure S1. Increase of crystallization peak temperature, $T_c$-$T_c^q$, of PLLAs during cooling at 10 °C/min caused by shearing at 170 and 150 °C versus shear rate, $\dot{\gamma}$. $T_c^q$ denotes the crystallization peak temperature of control specimens during cooling at 10 °C/min.

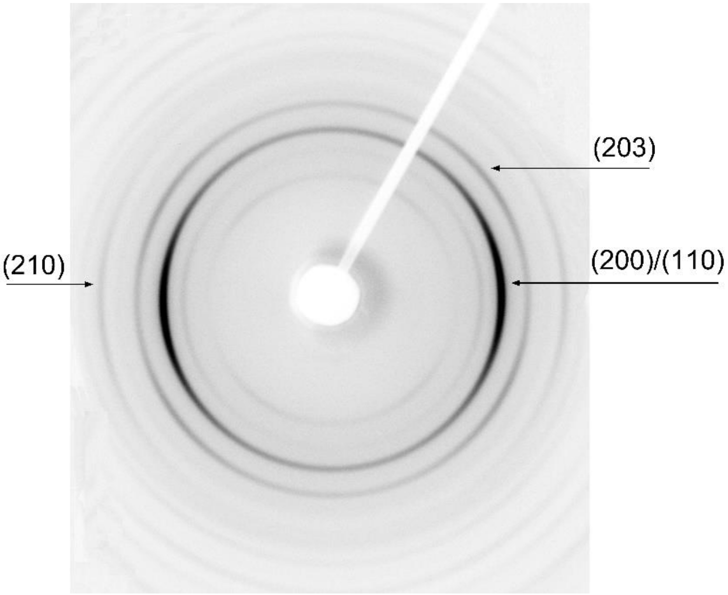


Figure S2. 2D-WAXS pattern of PLLA L339 sheared at 170 °C at 5 /s for 20 s and next cooled at 10 °C/min, with arrows indicating characteristic reflections.
